# Using digital technology to reduce drug-related harms: a targeted service users’ perspective of the Digital Lifelines Scotland programme

**DOI:** 10.1186/s12954-024-01012-y

**Published:** 2024-07-01

**Authors:** Graeme Strachan, Hadi Daneshvar, Hannah Carver, Jessica Greenhalgh, Catriona Matheson

**Affiliations:** Faculty of Social Sciences, University of Stirling, Stirling, FK9 4LA UK

**Keywords:** Drug-related deaths, Harm reduction, Digital inclusion, Digital technology, Digital health, Qualitative research, Scotland, Substance use, Connection, Person-centred care

## Abstract

**Background:**

Deaths due to drug overdose are an international issue, causing an estimated 600,000 global deaths in 2019. Scotland has the highest rate of drug-related deaths in Europe, with those in the most deprived areas at greater risk than those in affluent areas. There is a paucity of research on digital solutions, particularly from the perspective of those who use drugs who additionally access harm reduction and homelessness support services. The Digital Lifelines Scotland programme (DLS) provides vulnerable people who use/d drugs with digital devices to connect with services.

**Methods:**

This paper reports on the evaluation of the DLS from the perspective of service users who accessed services for those at risk of drug-related harms. A mixed methods approach was used including an online-survey (*n* = 19) and semi-structured interviews (*n* = 21). Survey data were analysed descriptively and interview data through inductive coding, informed by the Technology, People, Organisations and Macroenvironmental factors (TPOM) framework, to investigate the use, access, and availability of devices, and people’s experiences and perceptions of them.

**Results:**

Most participants lived in social/council housing (63.2%, *n* = 12), many lived alone (68.4%, *n* = 13). They were mainly over 40 years old and lived in a city. Participants described a desire for data privacy, knowledge, and education, and placed a nascent social and personal value on digital devices. Participants pointed to the person-centred individuality of the service provision as one of the reasons to routinely engage with services. Service users experienced an increased sense of value and there was a palpable sense of community, connection and belonging developed through the programme, including interaction with services and devices.

**Conclusions:**

This paper presents a unique perspective which documents the experiences of service users on the DLS. Participants illustrated a desire for life improvement and a collective and individual feeling of responsibility towards themselves and digital devices. Digital inclusion has the potential to provide avenues by which service users can safely and constructively access services and society to improve outcomes. This paper provides a foundation to further cultivate the insight of service users on digital solutions in this emerging area.

**Supplementary Information:**

The online version contains supplementary material available at 10.1186/s12954-024-01012-y.

## Background

Drug overdose deaths constitute a significant global burden, with an estimated 600,000 fatalities reported in 2019 [1]. North America has experienced a striking increase in drug deaths related to opioids [2]. In Canada, opioid-related drug deaths, including those attributed to synthetics such as fentanyl, are particularly problematic and were frequently found to be used in combination with other drugs when contributing to the 5,975 deaths registered from January to September 2023 [3]. In the USA in 2022 there were 107,081 fatalities identified as drug-related, [2]. Europe has also observed troubling drug death statistics with 6,677 deaths recorded in 2021, the majority of which were attributed to illicit opioids and synthetic alternatives [4].

Scotland maintains the highest rate of drug-related deaths in Europe and more than double the broader UK average reported in 2021 [5]. The average age of people who have died due to drugs has risen from 32 years in 2000 to 45 years in 2021, with most fatalities among long-term users living in the most deprived areas of Scotland [5]. Those in deprived areas are 16 times more likely to die as a consequence of their drug use than those in the most affluent areas [1]. In 2022 there were 1,051 drug-related deaths reported in Scotland, a reduction of 21% (*n* = 279) from the 1,330 reported in 2021 [6]. Although this reduction is welcome, there remains much work to be done and a long journey ahead before drug-related death is under control in Scotland.

In 2019, the Scottish Drug Deaths Taskforce (DDTF) was convened by the Scottish Government in response to the drug deaths crisis. The DDTF was made up of collective experts in the area, including those with lived experience. As a result of work across a three-year period, the DDTF developed an action plan containing 20 recommendations and 139 action points to be addressed by the Scottish Government to help continue to tackle the multiple challenges associated with Scotland’s drug death crisis [3]. Part of this work included the development of a person-centred, psychologically informed, human rights-based approach, placing those most affected at the heart of an evidence-based programme to address the drug-related deaths crisis [7]. The DDTF worked with the Scottish Government and other partners to introduce the Digital Lifelines Scotland (DLS) Programme, which considered the benefits of a digital approach and the provision of digital devices to vulnerable groups such as those leaving hospitals or prison or experiencing homelessness, for example, to remain socially connected and build relationships with services and thus reduce drug-related risks [8]. Additionally, the COVID-19 pandemic, and multiple challenges that society faced enabling human connection without contact further enhanced the focus on providing digital solutions for the most vulnerable.

Socially disadvantaged groups have the most difficulty fully accessing the benefits of digital services which creates a negative loop where those who are digitally marginalised are consequently socially marginalised and vice-versa [9].

The DLS programme sought to provide support, opportunities, education, and new, innovative approaches to create diverse and bespoke digital solutions targeted at service user welfare [9]. People at risk of drug harm are provided with digital devices which included smartphones, tablets, desktops and laptops for personal use, depending on need and availability. Data and access to these devices were provided independently by each service provider who procured their own devices. Devices could be used for any reason suitable to the service user, including making calls to family and accessing college courses. It follows the values of the Scottish Approach to Service Design which has a participatory, inclusive, and non-judgmental approach to acknowledging multiple, varied, and diverse service user needs [10]. The need to understand service user problems using a person-centred approach, by listening to those at the heart of the issue, improves the opportunity to cultivate solutions using this co-design strategy [9].

The “Early Adopters 1” (October 2021 – May 2022) [11], initiative of the DLS programme reached out to community groups in both urban and rural areas with grants to assisted in providing crucial access to peer and societal connection through the digital devices as well as access to greater harm reduction information on solutions such as naloxone, Medication Assisted Treatment (MAT) standards, and overdose response [5]. The inclusion of service users or people experiencing marginalisation within research has been used to great effect in many previous studies showing the importance of engaging with service users as contributing partners who share their unique lived experiences [12–14]. This level of inclusion allows services and programmes to be developed and tailored, potentially leading to relevant individual and collective community benefits [14, 15].

Early Adopters 1 provided a platform for the subsequent “Early Adopters 2” (May 2022 to February 2023) which further developed this initiative and focused more on those considered critically vulnerable such as those leaving hospital, exiting residential care or prison, or experiencing homelessness [9]. DLS will continue to fund new and existing initiatives and work streams until at least 2025 [9].

The DLS builds upon previous international research that has investigated digital platforms to improve overdose prevention among harm-reduction community organisations using both harm-reductionists and people who use drugs [13, 16–18]. A qualitative study conducted in Texas, USA, indicates an appetite for harm reduction-based digital solutions and platforms that would assist those who are using drugs to access information and services, and help to tackle their current overdose crisis [13]. Implementing a user-centred design, 20 harm reductionists and 24 people who use drugs participated in video interviews regarding the barriers to reporting overdose, what would help people report more overdoses, and how opioid-related incidence could be tracked among third sector and community organisations. Issues such as digital privacy and confidentiality were described as barriers to engagement and participants suggested employing trusted and respected community organisations to engage with service users and promote digital communication with people who use drugs [13]. This would encourage better reporting of overdose due to these long-standing and credible relationships between service users and community organisations [13]. Collective access to digitally connected records was accessible by all community organisations for a rapid and improved overdose response [13]. Use of digital technology to alert users of potentially dangerous street supply and cluster areas while recommending vigilance when buying drugs was suggested [13]. Additionally, instances of overdose or knowledge of precarious levels of strength or quality in the street drug supply through digital means were also viewed as beneficial due to this burgeoning evolution in technology in the harm reduction field [13, 16].

Moreover, in Vancouver, Canada, findings from the 2021 British Columbia Harm Reduction Client Survey highlight the impact of engaging with community harm reduction providers and the trust that develops to allow this type of digital communication to be mutually respected as a credible harm reduction measure [16]. 62% (*n* = 300) of participants reported that they had received drug alerts or engaged with a friend or peer when receiving this information [16]. Moreover, 67.4% (*n* = 261) of those who responded to a question on behaviour change indicated they had changed their behaviour positively because of this digital harm reduction alert, and 32.6% (*n* = 85) indicated they were impervious [16]. The study highlighted the trust and credibility that digital solutions can provide and indicated that more research on digital alerts and how they could assimilate with social networks, differing demographics and methods of communication would be beneficial [16].

Digital inclusion, and the opportunity to connect and remain connected to families, friends, and support services has been historically problematic for people who use drugs due to the paucity of finance, knowledge, and confidence in the area, plus many people’s often transient lifestyles [19]. However, since the COVID-19 pandemic, there has been increased focus on digital communication, highlighting a critical need for digital inclusion for those who are at risk of drug-related deaths to connect with services, stay safe, and have the opportunity to participate in broader society [20, 21].

Digital inclusion is the practice of ensuring equitable access to digital technologies and the internet, aiming to bridge the digital divide and enhance social and economic opportunities for all. It involves addressing various dimensions, including access to hardware, software, internet connectivity, digital literacy, and the ability to use technology for meaningful participation in society and the economy [22, 23]. In the context of drug-related harm reduction, the DLS programme was intended to provide, digital inclusion to help address the challenges faced by people who use drugs by providing access to critical information, support services, and opportunities for social connection, all of which can contribute to reducing the risk of overdose and improving overall well-being.

More critically, harm reduction research from Scotland has suggested that the new digital expectations around contact and the absence of face-to-face services during the COVID-19 pandemic provided barriers to previously successful harm reduction services [24]. Some participants voiced concerns regarding new digital anxieties, but others were more enthusiastic, noting better access to services and feeling more in control [24]. Moreover, recent scoping research suggested that although digital technology is viewed by some with apprehension and suspicion, the COVID-19 pandemic demonstrated the potential benefits of access to advanced digital services [20]. The DLS programme aims to address these challenges by providing digital solutions and promoting digital inclusion for those most vulnerable to drug-related harm.

Between 2022 and 2023, the DLS programme was evaluated, using surveys and semi-structured interviews with those receiving services, those delivering services, and the wider programme team, to understand the impact of the initiative.

This paper draws on qualitative and quantitative data from service users only, to explore their experiences of the DLS programme through the provision of devices (e.g. prepaid smartphones, tablets and internet dongles with internet data connection), or other digitally delivered support to reduce drug-related harm. By focusing on the perspectives of service users, this paper aims to contribute to the current understanding of how digital solutions can be effectively leveraged to address the challenges faced by those most vulnerable to drug-related harm. The findings from this study will help inform future initiatives and policies aimed at reducing drug-related deaths, particularly in the Scottish context.

## Methods

This paper reports the findings of a broader evaluation of the DLS programme using factors of the Technology, People, Organisational and Macro-environmental (TPOM) framework which had previous success in related research exploring the adoption of health information technology solutions [25, 26]. This framework was chosen for its ability to guide change implementers through both formative and summative evaluations, thereby aiming to prevent unintended consequences by addressing all facets of a project. Originating from the UK and informed by national programmes, this user-friendly framework presents specific questions for each of its four dimensions, intended to be considered at different points during a change programme. This paper will focus predominantly on the People and Organisational domains of the TPOM, with limited but crucial reference to the Technological domain. This framework was utilised to design research questions and analyse the qualitative data. Ethical approval was granted by the University of Stirling’s General University Ethics Panel (GUEP, 7799), the Ethics Subgroup of the Research Co-ordinating Council of The Salvation Army, Turning Point Scotland, and Shine Mentoring. All participants provided informed consent via electronic, written, or verbal means.

A mixed methods approach was employed for the study which included a quantitative survey and qualitative interviews [27]. The use of both a survey and interviews provided a means of maximising inclusion and was designed to be complementary. This approach was chosen based on preliminary findings from the survey, which suggested that a purely quantitative or qualitative approach might not capture the complexity of the issues at hand. By combining both methods, the study aimed to provide a more comprehensive understanding of the topic. The survey provided an overview of the client group and their access to digital devices whilst and the interviews allowed more nuanced detail on how devices were used. Service users were eligible for participation if they had used unregulated street drugs during the previous 12 months and were in receipt of a digital technology-based innovation services or devices that were funded by the DLS programme, or they had been offered such technology but refused (interviews only). Exclusion criteria were people aged under 18 years; unable to provide informed consent; unable to speak/understand English; severe mental health or behavioural problems or under the influence of substances; not currently living in Scotland; and not involved in the DLS programme.

### Survey data collection and analysis

The online survey collected structured descriptive data which was complementary to interviews. It was offered as an alternative for those who did not want or could not participate in interviews (e.g., due to time restrictions), although some people opted for both survey and interview. The approach to the online survey with this population, utilising cooperation from stakeholders and partners, and using multiple avenues to communicate, had been successful in previous studies [28].

The online survey was created using the JISC online platform. Participants could access the survey from August 1st 2022, to February 13th 2023. It was distributed online via an e-mail link sent to all organisations which received DLS programme funds, and service providers were asked to share the link with service users to complete electronically. Initial engagement with the survey was low, therefore, members of the research team met with service providers to answer any queries from service users, resulting in an 84% increase in engagement. According to the number of smartphones distributed (*n* = 120), a minimum of 15% of people who were engaged with the programme responded to the survey. Various reasons, including limited time for conducting data collection, not providing payment vouchers, and indirect access to users, were cited as contributing to the low number of responses. As the DLS programme involved the provision of digital technology, an online survey was deemed most suitable.

Informed consent was obtained via a tick-box before survey commencement, with support information provided afterward. Participants were asked not to disclose personal information with which they could be identified. In addition to demographics and health status, the online survey covered current use of technology, digital literacy, the type of technology already used, access to devices and support services, type of device used, frequency of use, method of use, reason for use, skill levels and training and support needs (Supplementary file 1). Survey results were downloaded from the online survey platform and used to generate tables. Data were downloaded to Excel by HD and formatted to provide an accurate quantitative illustration of the results. Basic descriptive statistics were used to describe the findings. Free text responses were themed in Word. This was completed by GS who used free hand thematic analysis for a more bespoke and personal approach to the less extensive qualitative data that was provided from the free text question for one response in each of the individual quantitative surveys.

### Interview data collection and analysis

The interview schedule (Supplementary file 2) was developed by the research team, covering the relevant domains of the TPOM, and informed by the broader DLS programme delivery work and a previous user needs study [8, 20]. The topic guide for service user participants focused mainly on the social/human (People) domains by exploring how the technology was used and the impact on service user and relationships with service providers.

All organisations who received funding from the DLS programme were provided with information about participating in an interview by email and asked to share this with relevant service users. The service providers worked with the research team to identify relevant participants and to arrange interviews with them. Interviews were conducted by phone, online, or in person. Written or verbal consent was sought from all service users prior to each interview. Interviews were conducted by GS, HD and JG. GS was a postgraduate researcher with extensive lived experience in multiple areas related to drug use; HD was an experienced research fellow; and JG was also a postgraduate researcher.

Interviews lasted an average of 17 min (range 6–30 min). All interviews were audio-recorded with permission. Researchers made reflective notes after each interview to cover contextual information of relevance including quality, substance, and any meaningful details unconnected to the study. All participants were provided with debriefing sheets at the end of the interviews and a £10 shopping voucher as an honorarium. The research team also attempted to interview service users who had been offered digital innovations via the DLS programme but declined, but no individuals were forthcoming.

Interviews were transcribed in full by an external transcriber and any identifiable information was removed. Transcripts were uploaded to NVivo (version 12) software. Thematic analysis using a social constructionist approach was employed to identify themes within the broad domains and sub-domains of the TPOM. This supporting inductive approach allowed for additional associated themes to be developed within the TPOM structure [25, 26, 29]. GS coded three transcripts to develop the initial coding framework, which was reviewed by HC. No structural changes to the coding framework were identified and GS completed coding of the remaining transcripts. The data from all interviews were initially coded and then data from service users only was arranged into themes and sub-themes for this paper. Quotes were pseudonymised and attributed to each participant; identifiable names, places, or people were removed.

## Results

### Survey results

Nineteen service users completed the survey: 13 men, four women, and two people who did not specify gender. There was a propensity for social/council housing (63.2%, *n* = 12), many lived alone (68.4%, *n* = 13) and had attended high school/college (94.8%, *n* = 18). They were also mainly over 40 years of age (78.9%, *n* = 15) and lived in a city (68.4%, *n* = 13). There were 94.7% (*n* = 18) who identified as having long-term physical and mental health conditions and almost three-quarters, 73.7% (*n* = 14), possessed school-level education, and just over one-fifth, 21.1% (*n* = 4), had attended college. Table 1 provides participant demographics.

**Table 1:** Participant demographics including accommodation, education and living situation (*n* = 19)

Type of current accommodation	Number	%
I own my home	1	5.3
Private rented	3	15.8
Council / Housing Association / Social Housing	12	63.2
With family/friends	3	15.8
**Living situation**		
Live alone	13	68.4
Live only with partner	3	15.8
Live with wider family members	0	0
Live with people not related to	1	5.3
Prefer not to say	2	10.5
Other	0	0
**Education level**		
School	14	73.7
College	4	21.1
University	0	0
N/A	1	5.3

Phones were the most popular device with 100% of those who responded (*n* = 18) indicating they could access a smartphone. The second most popular was a tablet, with 50% of responding (*n* = 9) participants having access and there were also mentions for laptops, desktops, watches, and voice assistants. The majority had daily connections to the internet on their phones (84.2%, *n* = 16) with 57.2% (*n* = 8) having a constant home internet connection. Contacting family and friends was the most prevalent use for devices with 70.6% (*n* = 12) using text messages, 43.8% (*n* = 7) using social networking, and 22.2% (*n* = 4) using video calls. Table 2 provides an overview of the use of technology.

**Table 2:** Use of digital technologies to connect with family and friends

Method	Everyday		Few times a week		A few times a month		Less often		Never	
	*N*	%	*N*	%	*N*	%	*N*	%	*N*	%
Video call (*n* = 18)	4	22.2	7	38.9	1	5.6	1	5.6	5	27.8
Text message (*n* = 17)	12	70.6	5	29.4	0	0	0	0	0	0
Social networking (*n* = 16)	7	43.8	6	37.5	0	0	0	0	3	18.8
Email (*n* = 17)	2	11.8	9	52.9	3	17.6	3	17.6	0	0

Additionally, devices were routinely used for health and social needs with over two-thirds, 68.4% (*n* = 13), highlighting the use of these services. Internet searches were accessed daily by 35.3% (*n* = 6) and 63.2% (*n* = 12) indicating they searched for information or help on drug use. However, 23.1% (*n* = 3) preferred face-to-face contact and were wary of digital technology due to privacy and broader societal trust issues. Other data highlighted a need to build confidence (38.5%, *n* = 5), and trust (23.1%, *n* = 3) in using digital technology. In addition, cultivating users’ knowledge of digital technology and its benefits was emphasised by over half of those engaged (53.8%, *n* = 7). Half (50%, *n* = 9) were also interested in refining their basic computer skills (18 replies) and 52.9% (*n* = 9), considered a good understanding and proficiency using the Internet to be the most valuable (17 replies).

### Interview results

Twenty-one interviews were conducted with service users, with one being subsequently withdrawn due to concerns over the participant’s vulnerability. The findings are presented as three themes, related to the ‘Technological’, ‘People’, and ‘Organisational’ domains of the TPOM and the subordinate sub-themes that evolve from them.

### Technology

Three sub-themes were identified in relation to ‘Technology’: cultivating connections; lack of technical knowledge; and usability as a key enabler or barrier of digital technology.

#### Cultivating connections

The advantages of digital technology enable individuals to connect with others, including friends, family, and support services. This connectivity offers a wide spectrum of support, from combating feelings of isolation and renewing connections with family and services to forming new friendships and improving self-esteem. This participant demonstrates the salience of accessible connection to others through the provision of a digital device:

*Aye [yes]. No no, because before I got the iPad and that, I was just… I wasn’t really connecting with people, if you know what I mean. It made me feel more connected with people.* (Participant 1)

Another participant emphasises that access to devices and social media has improved their situation and contact with their social worker:

*It makes me feel much better because Facebook and all that, looking up things as well, and also with my like support worker kind of like sometimes emails me… or he’ll sometimes send me on my Facebook “right I am here, come and get me”, whatever. He has, if he can’t get hold of me, he will like just like text me or something.* (Participant 14)

This participant highlights the benefits and positive impact of potential connection with their mother and the emotional context involved with their mother, even though they are in poor health:

*But I’d love to just phone my ma [mother] again and be able to see her [daughter], for her to see me, that I’m a’right [alright]. I look really terrible now because I’m no [not] well an’ that, but….* (Participant 13)

However, for others, digital devices already provide a conduit to family and friends that would otherwise be inaccessible:

*Yes, I keep in touch with my daughters, I’ve got four daughters, I am only in touch with two. So, I keep in touch with my two daughters that I’m in touch with and obviously like my friends, my family, kind of things like that.* (Participant 14)

Participants openly discussed the significance of digital devices and the impact they have made, or could make, in improving connections and accessibility to family members in particular.

#### Lack of technical knowledge and access

The lack of technical knowledge and access was underlined by participants when navigating the intricacies of new devices. Their previous experience involved basic and inexpensive analogue phones without internet provision. Furthermore, they suggest a potential fear of technology and historical lack of access but there is support and encouragement from others who are facing similar challenges:

*Aye [yes], yeah, there’s a lot of women all together, it’s no [not] just me. I thought I was the only technophobe in here [laughs]. But there’s other women in here, they’ve just been used to having like the wee £10 Alcatel phones and stuff like that, wi [with] no internet access. So, we’ve kinda, there’s one or two o’us that arenae [aren’t] very good at it, so I mean we’ve been able to like just help each other out, and like send like picture messages to each other and do video calls and stuff like that.* (Participant 9)

Additionally, participants highlighted how a lack of access to the internet was addressed through connection to the online resources provided by the programme. It improved their confidence due to the help and advice offered by staff members:

*They want to like send you stuff through and that, and I’ve got that, I couldn’t access that because I didn’t have any internet. So that’s been good, you know what I mean. And then [staff member] and [staff member] showing me, giving me the confidence, how to look up things, how to do that. That’s really helped.* (Participant 9).

*I had support with how to use it and that. The person who came out and gave me the laptop set it all up for me and gave me support for it. I got support they gave me. It’s just difficult starting off and getting it all set up and that. But once it was set up it was fine really, as long as they set it up for you and show you what to do, kind of thing. How to go into… and kind of within me, but once I got used to it, it was alright.* (Participant 2)

Participants talked about the difficulty encountered when initially receiving their digital devices but were happy to receive the support of staff when installing the laptop and were then able to navigate the remainder of the learning process.

#### Usability as a key enabler or barrier of digital technology

Although benefits associated with technology provision were realised, the usability of the different devices, digital technologies, and applications was varied, depending on individual circumstances and experience of technology. The benefits of online communication using technology (such as tablets) were highlighted as it assisted in engaging with a broader range of people:

*Basically, because I couldn’t get Zoom on my phone but I can do it with the iPad, so the iPad’s helped me communicate with a wider, with people from [location], people from [other location] and everything like that, so it’s getting me connecting with other people.* (Participant 19)

Additionally, access to educational devices such as a computer and the learning that they have been engaging with at college were described as beneficial:

*I’ve been learning on an actual computer rather than a thingummy [tablet], you know what I mean, like folder and all that and saving all my stuff in it. I’ve been learning how to do that at college.* (Participant 17)

These participants both expressed different preferences and feelings and benefits associated with devices offered by the programme. They indicate that some devices are preferable to others, depending on individual predilection, situation, challenges or perhaps educational ambitions.

### People

A number of themes were identified within the People factor. These were: digital technology as a connection to community; data privacy; apprehension around engaging with digital devices; and individual support incentivises engagement.

#### Digital technology as a connection to improve wellbeing

Participants talked about digital technology as a vehicle to providing and creating connections, which would increase social cohesion, confidence, and relationships. This highlights the important connections made online, enhancing a feeling of community which can help marginalised individuals build confidence and make new connections when they meet in person:

*The day I kind of walked in the door at the community, I never really knew anybody. [Now] I know tens, do you know what I mean? Aye [yes], that is like you mentioned, the Zoom meetings and that, I get to know people that way and then when you meet them in person, you have kind of broken down a barrier.* (Participant 1)

Another participant talked about digital connection and the opportunity to engage with services that were previously problematic such as mental health:

*If you connect, that word keeps coming in, if you connect a lot better especially with mental health it’s a big thing the now. Especially for men. Men are embarrassed if they’ve got anything to do with mental health.* (Participant 19)

Similarly, participants also described how talking to and connecting with people through devices is crucial for their continued wellbeing and interaction with each other and society. Using the phone to ask for support is highlighted below:

*Well, if you’re feeling down or anything you can pick up the phone and speak to somebody. It’s good, cause you get to talk, no what I mean. So that is a good thing, you can get to talk… [if you’re] bothered by anything you can just talk about it.* (Participant 11)

Furthermore, this next comment suggests the previous lifestyle experienced was a contributing factor to the need to find support from others through digital connection:

*And you definitely need that, especially if you’ve been involved in my lifestyle. The one thing you need is people. And that’s one thing the iPad does offer, a way of connecting with people.* (Participant 4)

These comments indicate that digital connection through various devices has a positive impact on wellbeing. Improving confidence and access to peer support and services is, they feel, crucial for improving their lives.

#### Data privacy

Participants highlighted that service users regularly feel unfairly scrutinised, judged, and stigmatised by society. There appears a deep-rooted unease when engaging with mainstream society and how service providers or stakeholders approach service user engagement, such as research projects, may be viewed as disingenuous or even nefariously motivated. This was evident in particular around the use of people’s data. Service users were apprehensive about providing any location or privacy information; speculating that this data would be used to monitor or covertly oversee their behaviours:

*I was thinking myself, well, can you see where I am all the time? Or stuff like that. That and all just ways you can, I don’t know, I don’t really know if there is anything gaun [going] on behind the scenes about data or whatever… There has been women that have said that “oh, is that just to check up on us?” So, a lot of women start thinking, “Well, why are we getting these phones? Is that to keep tabs on us?“.* (Participant 9)

Concerns were also raised over personal information being misused, however participants did not specify by whom:

*I don’t see the point to it. All you’re going to do is access people’s personal information and just use it against them.* (Participant 7)

Participants were apprehensive around data privacy, and it being shared with others for purposes out with the remit of this study. This particular cohort live lives on the margins of society where suspicion of people, places, and things is ever present. Without reassurance and guarantees that data privacy will be respected, engagement with these devices would be difficult.

#### Apprehension around engaging with digital devices

Participants highlighted a fear of the unknown and a lack of knowledge and confidence in using digital devices. This may cultivate a propensity to disengage from any benefits of digital technology that are in conflict with a life living in the shadows or more explicitly off the grid:

*Well certainly we know not everybody wants to access the digital world and now I don’t know if that’s the fact that they want to be off the grid and they don’t, they understand digital and don’t enjoy it and choose not to. Or it may be that there’s a fear of the unknown and just say they don’t want to because they don’t know how to work [it] or maybe don’t understand the benefits it can bring as well.* (Participant 12)

This suspicion of digital technology can be attributed to a number of reasons, for example, due to unfamiliarity with digital devices, sceptical of the benefits available or wishing to be ‘off the grid’ [30]. Some participants were more vocal when conveying their preference to engage face-to-face rather than through an online medium:

*Aye [yes]… in person but I would do it online as well, but it is better in person, definitely better in person because you are face to face with that person. I mean, that’s just my thoughts anyway, I would rather do it in person but doing it like this, I don’t like doing it like this, but I don’t mind.* (Participant 3)

This participant highlights the importance of retaining the option of providing face-to-face contact with those who are marginalised or vulnerable. They appear to indicate their belief that it is a more effective method of engagement.

#### Individual and collective support incentivises engagement

Person-centred incentives and support, which promoted individual solutions, were welcomed by the participants due to their diverse and distinctive challenges. Some participants appeared impassioned over their interactions with, and support received from staff. The staff/service user relationships provided the greatest assistance to digital use both personally and socially. This participant suggests that person-centred support incentivised engaging with services and using digital technology. There was motivation to learn and to return to engage further with service providers:

*Yes, the staff in the hub, if you need any help with anything, you come in. Maybe one specific member won’t know but there’s always going to be somebody that will be able to help you, be it with your emails, be it with downloading something, be it with just using and setting it up. I’ve seen the staff helping each other in here with things.* (Participant 15)

Other service users highlighted both interactions with community staff and collective peer support and friendships important to their engagement with services:

*Well, a lot of the workers, I know a lot of the workers in here, and I get a lot of support … It’s brilliant in here. (Participant 8)*

The camaraderie and support are further described below with the reacquaintance of the participant and a former friend. They state that they, along with new friends in the group, get on very well with each other:

*Aye [yes], I’ve met a lot of new lassies [women], like friends. And a lassie [woman] that know that used to stay in [Location] when I stayed there years ago, I met her again. And it’s good to see her after 20-odd years, d’you know what I mean. And then like I’m pals [friends] with a lot o’ [of] the lassies [women] now, and we all get on brilliant, so really good.* (SU8).

These participants have highlighted the individual and collective support received from staff and fellow service users as a crucial tenant of their continued engagement with both physical and digital services.

### Organisational

People and organisational factors appear to enjoy a synergetic overlay. The two sub-themes in the Organisational domain have similarities with those on the People domain. These sub-themes are digital and personal support, and harm reduction with access to digital services.

#### Digital and personal support

Service providers played a key role in digital uptake. The friendly and genuine interest in service users was critical to ensure a welcoming, community environment:

*[Service] definitely needs to be here. It is the heart of the community, like as in the homeless community. And it’s, it’s so unusual, pure joy, because it’s like… when you’re lost, and you can just chat to somebody. You’ve always got someone to bond wi [with] in here.* (Participant 7)

Service providers were perceived as creating a safe space to feel at home, who respect and understand participants’ daily struggles. These frontline communities provide a mixture of trusting and helpful services, camaraderie, and support from peers:

*So, we’re doing a lot of things with the women’s group just now that are digital, and it’s really like you can phone, you can look up things, and get a bit o’ [of] support. Like we’re doing meetings, drug meetings and stuff like that, cause you can go onto Zoom and do this for various meetings and stuff like that. So it’s instead of coming in here for support all the time, even though I have got a worker, if I’m like finding dealing with stuff fae [from] court and having a really bad day and stuff like that, I can like look, see what meetings are online, try and join something, and just try to keep connected wi [with] people who are going through the same kinda [kind of] thing*. (Participant 9)

Taking the time to introduce service users in a way that builds confidence in digital services and devices has really helped the participants. Furthermore, it appears blending personal support with access to digital devices and connection is providing a positive community experience.

#### Connecting through harm reduction and access to digital services

Person-centred harm reduction benefits were evidenced here through access to group support and digital services for service users which provided connection and a sense of improved wellbeing. This participant stated they were attending a harm reduction course and enjoyed the focus on finding individual solutions. They are also using this to help connect with others. In this case, harm reduction appeared to enjoy a concomitant synergy with digital solutions as they both complement each other tackling the potential for negative outcomes and improving self-esteem:

*That was when I’ve been going to the women’s harm reduction course. But I’m going to the [service provider] course as well. So, that’s going to be good as well because they said they’re gonnae [going to] do like things that’s reflecting different people’s outlook and stuff that’s happening… I’ve only started [with] the [service provider], but that will be good because I’ve got my data, and I know I can like connect with them as well.* (Participant 9)

This was further explored with the discussion on harm reduction apps and the potential for instant updates and access to information. Two participants felt that the synergy between harm reduction and digital solutions was a positive development. This first one is optimistic over the potential to provide instant messages for harm reduction through a phone:

*So it’s good if the service provider] are able to give them a phone. And then, as I said, we did that app. And that sends a message through on harm reduction, and let’s say for drug use, other misuse and stuff like that.* (Participant 14)

While this second one illustrates the mileage in providing this for the younger generation to enable a more harm reduction focused and empowered approach from them:

*It would be [harm reduction apps useful], especially the young generation at this point because there’s a lot of them don’t even have a clue when they’re taking ODs [overdoses] and all that. I’ve been there with the drink; I know what it’s like*. (Participant 9)

These two participants demonstrate an appetite for instant advice and access to information detailing potential issues with the local drug supply. These digital harm reduction methods provide instant information, support, and reassurance to service users.

Overall, data from all interviews suggested that connections, in multiple forms, were seen as crucial to personal progress. Device usability, ensuring suitability, acknowledging disability, harvesting knowledge, providing education, building confidence, and protecting privacy were all identified as key areas of concern.

## Discussion

This paper provides evidence of an appetite from service users to engage, shape, interact, and evolve through the structural paradigms of the DLS programme to improve both individually and collectively [31]. Participants discussed confidence, knowledge, inclusion, incentives, support, and privacy help to overcome barriers and enhance accessibility to digital solutions. Many participants lived alone, in council accommodation and had long-term health challenges which affected their physical and mental well-being but were still eager to engage with digital devices and explore the barriers to and benefits of digital inclusion through device.

This paper provided a focused account of the experiences of digital technology via the DLS programme. The findings highlight the benefits of, and an appetite for, the current DLS programme and the expansion of related services. Additionally, similar research where harm reduction services were broadly welcomed and appeared to enjoy a complimentary and synergetic relationship with remote digital services highlights the wider international appetite for digital solutions [13, 16]. More education and learning opportunities are required to ensure that service users feel included and acknowledged within society to reflect what they can offer through their experience and potential. Moreover, reassurance that personal data and interaction with services would remain confidential from macro-environmental agencies is critical [13]. One macro-environmental issue that remained latent within the discussion was how the access to digital devices, data, and services would continue to be financed considering how well they have been received. This was a very nuanced issue and one that remains so as the purchasing and financing of individual packages was left to individual partners and community stakeholders. In terms of how much, where, to whom, and for how long any future packages could be provided to individual partners depends on how funding agencies decide to distribute funding and the form that may take.

The connection has a potentially further unintended consequences regarding 4G/5G connectivity and the complexities as we digitally evolve into more ubiquitous 5G network coverage and included data plans, and how these will be provided with security, reliability, and regularity to service users. It will be crucial to ensure that digital solutions and access to technology are maintained following an end to funding awards. The Scottish Government should seek to ensure that provision remains accessible for these vulnerable individuals, including introducing a universal data allowance, provided by governments or through public and private partnerships, as a human right to every citizen, helping to tackle issues of sustainability [32, 33].

Interview participants highlighted increased connectivity and the opportunity to connect with friends and family through digital means. They also emphasised the benefits of digital technology when living alone, feeling isolated, disadvantaged, or disassociated from society, as has also been noted elsewhere [34]. However, despite this, participants in our study were concerned both with usability and technical knowledge related to devices which would assist their efforts to maximise the potential of the devices [19, 20]. Another barrier was individual learning needs, and there were suggestions that this could be tackled through the provision of appropriate devices and tailored support for vulnerable or marginalised users [35]. The apparent service user willingness to engage with digital devices offers personal and collective insight. This research provides a grounded and honest account of experiences and eagerness to continue to assist in encouragement of service users to continually engage in the shaping and creation of new services which aim to support digital inclusion. The findings presented here show that providing digital means can promote feelings of inclusion for vulnerable groups and shows how involving these communities within research to develop digital solutions can support decision making in a progressive way.

Data privacy was crucial as some participants harboured an instinctive and innate mistrust in how data were used, and scepticism over why devices were provided for free. The potential for covert observation, or the inability to conceal location, was crucially problematic for some participants. Considering the participant demographics, their experiences of societal marginalisation, and the deep mistrust of police, authority, and institutions [36], this is an understandable concern. In contrast, more progressive countries have enjoyed many years of harm reduction evolution and built long-standing trust in digital devices which has enabled initiatives such as instant electronic contact and messaging alerts [13, 16, 36]. This study supports previous research that indicated a fear of the unknown and a lack of self-esteem and confidence may create a propensity to avoid investigating the individual benefits that digital can provide [37], which is balanced by the acknowledgement that crucial connections, community, and relationships can be built through digital technology [37]. Furthermore, a person-centred, non-judgmental approach embraced by service providers was highlighted by service users as a motivation to continually engage with services which replicates previous finding from research in the USA [13]. Service providers and harm reduction community organisations taking time to build trust and rapport on a human level with service users and vulnerable cohorts appear to produce constructive outcomes for users and providers [16, 38–40]. This study’s results indicate that Scotland has a long way to go to convince some service users to embrace the new digital revolution; however, many others are encouraged by the progress and would welcome access to instant harm reduction messaging and information.

The data also reflects previous Australian-based literature suggesting interaction with digital services and harm reduction communities helped those experiencing homelessness find a sense of structure, safety, community and belonging via community and daily interaction via digital devices [37]. Harm reduction services were highlighted through some participants’ appetite for services and acknowledgement of the benefits of an individualised approach. There may be a desire by many service users to interact with providers, overcome challenges and embrace digital health solutions as a life-improving tool [39]. This same positive and overlapping interaction is also observed in recent community harm reduction research that examined a variety of harm reduction methods including community hubs, digital platforms, health-related outcomes and substance use [16, 38–41].

More broadly, the study findings support cross-discipline qualitative results connecting psychosocial patterns that show harm reduction benefits of digital inclusion with those who are marginalised, disabled or experiencing ill health in that digital harm reduction services can potentially enhance confidence, self-esteem, and the promotion of a collective self [42]. However, this should be tempered by a meta-analysis of substance use related to telemedicine and digital technologies conducted by Hamideh and Nebeker [43], who emphasised the need for caution and to prioritise a human-centred psychosocial approach to digital solutions [24, 43].

Harm reduction initiatives are not conceptually foreign in Scotland and have also previously been successful with similar populations and projects that investigated people, community, and substance-related challenges [36, 38, 40]. There was respect and understanding of service providers and the role they play in assisting service users, but also in a more human and socially cohesive role that provides scaffolding, psychological comfort and daily structure which should continue to be provided and further enhanced within frontline services [44]. This person-centred harm reduction approach promoting this digital service appears to have a positive impact by expanding and providing immediate health and housing assistance, and opportunities in emergency situations, and reducing the related costs.

Providing further continual support and digital access for service users will require structural and community support from macro-organisations [30, 45]. This will require the continued provision of devices, support, and opportunities to interact with the digital world for vulnerable service users while acknowledging the cohorts’ understandable reservations. Digital devices could be used to further expand streamlined access to housing and finance and crucial health amenities that service users find difficult to access due to their precarious and transient daily existence [30, 45]. However, we should acknowledge the multiple personal reasons for digital anonymity; therefore, a legacy face-to-face service would be essential at least in the meantime, for those who are unsuitable for, or suspicious of digital connectivity [39, 46]. The imperative of listening to the voices of individuals directly impacted by a programme cannot be overstated for both service providers and policymakers. Our study not only underscores the significance of this practice but also delves into the critical challenges and facilitators associated with digital inclusion among this demographic. By illuminating these factors, our findings provide valuable insights essential for informing effective strategies and policies in fostering digital equity.

### Strengths and limitations

This study included a range of participants across Scotland. It is the first of its kind to consider the potential of digital inclusion as a means to prevent the risk of drug-related harm. We were able to include data from a range of people with experience of drug use by using a survey (*n* = 19) and semi-structured interviews (*n* = 21). The abundance of qualitative data in particular from service users is a clear strength of the study. Limitations were experienced which included potential self-report bias through the surveys. Demographic information such as housing, health, schooling, and other data such as device and internet usage may not be representative of the larger population at risk of drug-related harm. There were strict eligibility criteria which included drug use within the last 12 months which may have meant some people were unable to participate. Due to the time limitations, some service providers who had received support from the DLS programme could not be included which consequently led to an over-reliance on service users attached to organisations which were able to disseminate devices more quickly than others. Crucially, this may lead to additional participant bias at a service level. Furthermore, the potential for service providers to pre-select favourable participants who were more digitally engaged was strong [47]. This was further compounded by the absence of a number of participants who were asked to contribute but declined. This may further bias the cohort due to a potential positive predilection for the digital inclusion project.

## Conclusion

The Scottish Government introduced the DLS programme to provide digital devices to vulnerable groups, aiming to enhance social connection and reduce drug-related risks. Digital inclusion is crucial, especially so during the pandemic, to connect those at risk of drug-related deaths with services and broader society. The DLS programme was evaluated to understand its impact on reducing drug-related harm.

Service users represent marginalised populations and struggle with physical and mental health issues with almost all receiving a minimum of school education with some attending higher education. Qualitative data suggests an appetite for improved communication and connections but this is tempered by apprehension around data privacy. Some participants felt that they benefited from help and support when the received devices and others mentioned the improvement in their wellbeing from having this immediate access to connection and communications. Harm reduction services, the usability of devices and continued personal and collective support were all welcomed and crucial to continued service user engagement. Future work should examine the positive digital avenues that can empower service users to embrace digital opportunities, solutions, and prospects to engage with society, build new connections, bridge old divides, and help improve long-term services while utilising a psychosocial approach. Taking encouragement from the apparent lack of service user prevarication during the interview; further exploration and study to assist service users in building new digital connections in society and cultivate a collective sense of ownership and responsibility within broader harm reduction initiatives should be encouraged.

## Electronic Supplementary Material

Below is the link to the electronic supplementary material.

Supplementary Material 1

Supplementary Material 2

## Data Availability

The datasets generated and/or analysed during the study are not publicly available. Individual privacy could be compromised if the dataset is shared due to the small sample involved.

## Abbreviations

DLS: Digital Lifelines Scotland
TPOM: Technology, People, Organisational and Macroenvironmental factors
DDTF: Drug Death Taskforce
PWUD: People Who Use/d Drugs
