# Correction: Using digital technology to reduce drug-related harms: a targeted service users’ perspective of the Digital Lifelines Scotland programme

**DOI:** 10.1186/s12954-024-01100-z

**Published:** 2024-10-22

**Authors:** Graeme Strachan, Hadi Daneshvar, Hannah Carver, Jessica Greenhalgh, Catriona Matheson

**Affiliations:** Faculty of Social Sciences, University of Stirling, Stirling, FK9 4LA UK

**Correction: Harm Reduction Journal (2024) 21:128**

10.1186/s12954-024-01012-y

Following publication of the original article [1], error noticed in the first line of the Abstract ‘Deaths due to drug overdose are an international issue, causing an estimated 128,000 global deaths in 2019. Scotland.’ and in the first line of the Background section “Drug overdose deaths constitute a significant global burden, with an estimated 128,000 fatalities reported in 2019 [1]. North America”, the value ‘128,000’ should have read as ‘600,000’.

The original article has been corrected.
