# Supplementary material for: Using digital technology to reduce drug-related harms: a targeted service users’ perspective of the Digital Lifelines Scotland programme
Source: Harm Reduct J. 2024 Jul 1;21:128. doi: 10.1186/s12954-024-01012-y (PMC11218389; doi:10.1186/s12954-024-01012-y)
Supplement: Supplementary file 1 — Supplementary Material 1 [file 12954_2024_1012_MOESM1_ESM.docx]

**Interview Topic Guide**

**Service Users/People Who Use Drugs**

1. Do you receive any digital services/devices from any service providers?
   1. If yes,
      1. What services/devices do you receive and how do you feel about it in terms of: how easy it is to use or learn to use as well as security and confidentiality of data, confidentiality, etc.
      2. When did you start receiving the services and did they change over time?
2. What benefits have you experienced from using digital technology so far?
3. Thinking about the devices mentioned above, what do you typically use these for e.g. communication, information access etc?
4. What worked well for you in terms of devices and/or digital services? What worked less well? Why?
5. Please tell us how you feel your digital skills/ confidence? Do you think these could be improved? How?
6. Have you been involved in getting digital services started or used more? At what stages and in what ways?
7. Do you think the changes to digital technology have changed the relationships between you and the service providers?
8. Is there anything else you’d like to add?
