# Supplementary material for: Using digital technology to reduce drug-related harms: a targeted service users’ perspective of the Digital Lifelines Scotland programme
Source: Harm Reduct J. 2024 Jul 1;21:128. doi: 10.1186/s12954-024-01012-y (PMC11218389; doi:10.1186/s12954-024-01012-y)
Supplement: Supplementary file 2 — Supplementary Material 2 [file 12954_2024_1012_MOESM2_ESM.docx]

**Evaluation Survey**

**for people who use drugs**

**PIS information.**

1. You are eligible to take part in the study if you:

- Are aged 18 years and over,
- Currently use or used street drugs in the past 12 months
- Currently living in Scotland.
- Received a digital technology-based innovation funded by the Digital Lifelines Scotland programme.

Does this describe you?

- - *Yes/no (go to the end of the survey)*

**Consent box that ticks that they meet eligibility criteria.**

1. Do you consent to the above statements?
   - *Yes/no (go to the end of the survey)*

**Online survey questions for people who use drugs/have used drugs in the past 12 months**

**<<Personal Information>>**

1. Where do you currently live? (Please tick one)
   - *City*
   - *Large town*
   - *Small town*
   - *Rural area*
2. What type of housing or accommodation do you live in? (Please tick one)
   - *I own my home*
   - *Private rented*
   - *Council accommodation*
   - *Homeless hostel*
   - *With family/friends*
   - *Currently rough sleeping*
   - *Other (specify)*
3. Do you have any long-term physical or mental health conditions?
   - *Yes* □ *No*

□ *If yes, do you use digital technology to access information or services regarding your condition(s)? (This could include website/online chat/online constancy/ online prescription etc)*

□ *If you don’t currently, would you like to use digital technology to get help and support with your condition(s)?*

**<<Digital literacy>>**

1. Do you own/have access to / use any of the following devices?
   (Please tick the boxes that apply).

|  | I own | I have access to | I regularly use | I don’t have access | This device can connect to the Internet |
| --- | --- | --- | --- | --- | --- |
| Smartphone | □ | □ | □ | □ | □ |
| Desktop computer (PC) | □ | □ | □ | □ | □ |
| Laptop computer | □ | □ | □ | □ | □ |
| Tablet, iPad | □ | □ | □ | □ | □ |
| Smartwatch | □ | □ | □ | □ | □ |
| Voice assistance (e.g. Alexa/Google Home, Siri) | □ | □ | □ | □ | □ |

- - *Other* (Please explain – Free text)

1. Please tell us about your skills in using digital technology. Tick one box to show how much you agree with the following statements

| **I am confident that I can . . ..** | **Agree** | **Neutral** | **Disagree** | **Not Applicable** |
| --- | --- | --- | --- | --- |
| Log into PC or laptop | □ | □ | □ | □ |
| Send and receive emails | □ | □ | □ | □ |
| Share documents online with others e.g. sending documents as attachments | □ | □ | □ | □ |
| Download and save documents from the Intranet | □ | □ | □ | □ |
| Use social media | □ | □ | □ | □ |
| Use online banking, pay bills | □ | □ | □ | □ |
| Find relevant information using the Internet | □ | □ | □ | □ |
| Understand internet safety and security | □ | □ | □ | □ |
| Understand the principles of online confidentiality and data protection | □ | □ | □ | □ |
| Participate in video calls | □ | □ | □ | □ |
| Easily find online learning resources to help me in my treatment | □ | □ | □ | □ |
| Other | □ | □ | □ | □ |

(Free text box for free)

1. Which of the following ways do you connect to the Internet? (Please tick all that apply).

|  | How often do you connect to the Internet this way? | | | | |
| --- | --- | --- | --- | --- | --- |
|  | Every day | A few times a week | A few times a month | Less often | Never |
| On my mobile phone | □ | □ | □ | □ | □ |
| Home connection | □ | □ | □ | □ | □ |
| Work or college connection | □ | □ | □ | □ | □ |
| Public Wi-Fi internet | □ | □ | □ | □ | □ |
| Cafe Wi-Fi | □ | □ | □ | □ | □ |
| Library Wi-Fi | □ | □ | □ | □ | □ |
| Bus or train Wi-Fi | □ | □ | □ | □ | □ |
| Service provider computers/Wi-Fi | □ | □ | □ | □ | □ |

Other (please specify)

1. Which of the following do you use to connect to your family or friends? (Please tick all that apply)

|  | How often do you connect to friends / family this way? | | | | |
| --- | --- | --- | --- | --- | --- |
|  | Every day | A few times a week | A few times a month | Less often | Never |
| Video call (Zoom, Skype, Facetime, WhatsApp, MS Teams…) | □ | □ | □ | □ | □ |
| Text (SMS, WhatsApp) | □ | □ | □ | □ | □ |
| Social networking (Instagram, Facebook, Twitter, TikTok etc) | □ | □ | □ | □ | □ |
| Email | □ | □ | □ | □ | □ |

*Other (please specify)*

**<<Current Situation>>**

1. Which kind of digital devices have you received from the Digital Lifelines Scotland programme?

|  | How have you used it so far? | | | |
| --- | --- | --- | --- | --- |
|  | Call | Internet | Text messaging | other |
| Smartphone | □ | □ | □ | □ |
| Tablet | □ | □ | □ | □ |
| Internet connection | □ | □ | □ | □ |
| other | □ | □ | □ | □ |

1. Which of the following do you use to keep in touch with your service providers for support? (Please tick all that apply)

|  | Which devices do you use to do this? | | | | |
| --- | --- | --- | --- | --- | --- |
|  | Smartphone | Desktop computer | Laptop | Tablet | None of these |
| Video call (e.g. Zoom, Skype, Facetime, MS Teams, Google Meets etc) | □ | □ | □ | □ | □ |
| Text message (SMS, WhatsApp) | □ | □ | □ | □ | □ |
| Social networking (Instagram, Facebook, Twitter, TikTok etc) | □ | □ | □ | □ | □ |
| Online chat functions (Support organisations, Recovery Forums) | □ | □ | □ | □ | □ |
| Email | □ | □ | □ | □ | □ |

*Other (please specify)*

**<<Digital Technology and Services>>**

1. Do you use devices or digital technology to find out how to get help with health or social problems?
   - *If yes, what technology do you use? And how often?*

|  | How often do you do this? | | | | |
| --- | --- | --- | --- | --- | --- |
|  | Every day | A few times a week | A few times a month | Less often | Never |
| Search on the Internet | □ | □ | □ | □ | □ |
| Ask friends/family/support workers to search on the Internet | □ | □ | □ | □ | □ |
| Check the service provider’s website | □ | □ | □ | □ | □ |
| Check NHS website | □ | □ | □ | □ | □ |
| Check social media (Facebook groups) | □ | □ | □ | □ | □ |
| Forums | □ | □ | □ | □ | □ |
| Ask voice assessment (Alexa, Google Home, Siri) | □ | □ | □ | □ | □ |
| Contacting health care providers e.g. GP, Addiction service | □ | □ | □ | □ | □ |

- - *If not, why do you not use digital technology?*

*□ Please explain (Free text)*

1. When you need information about seeking help for problems related to drug use, do you use a device/digital technology? □ Yes □ No
   - *If yes, what technology do you use? How often?*

|  | How often do you do this? | | | | |
| --- | --- | --- | --- | --- | --- |
|  | Every day | A few times a week | A few times a month | Less often | Never |
| Search on the Internet | □ | □ | □ | □ | □ |
| Ask friends/family/support workers to search on the Internet | □ | □ | □ | □ | □ |
| Check the service provider’s website | □ | □ | □ | □ | □ |
| Check NHS website | □ | □ | □ | □ | □ |
| Check social media (Facebook groups) | □ | □ | □ | □ | □ |
| Forums | □ | □ | □ | □ | □ |
| Ask voice assessment (Alexa, Google Home, Siri) | □ | □ | □ | □ | □ |
| Contacting health care providers e.g. GP, Addiction service | □ | □ | □ | □ | □ |

- - *If not, why do you not use digital technology?*
    - *Please explain (Free text)*

1. Do you experience any difficulties in using devices or digital technology? *□ Yes □ No*
   - *If yes, please tell us about these difficulties.*
     - *I do not know how to use it*
     - *I don’t have enough confidence to use it*
     - *Do not trust digital technology*
     - *Privacy worries*
     - *Too expensive*
     - *Bad experience with viruses*
     - *Not for people my age*
     - *Other (please specify)*
2. Do you think having support to help you use devices and digital technologies would be helpful?
   E.g., training, skills development, confidence building, support? *□ Yes □ No*
   - *If yes*, *what might be helpful (tick all that apply and please add further ideas of your own)?*

*□ Having my own device*

*□ Easy to use instructions*

*□ Better access to the Internet*

*□ More support*

*□ Other (please specify)*

- - *If you don't need any support to help you use digital technology, please tell us why.*

1. Please tell us what digital technology training you would welcome

|  | Most Important | Quite Important | Not  Important | Not  Applicable  I can do this  already. |
| --- | --- | --- | --- | --- |
| Getting started with computers (Logging onto computer, keyboard and mouse skills) | □ | □ | □ | □ |
| Computing basics (word processing, emails, setup Wi-Fi) | □ | □ | □ | □ |
| Using the internet (searching, etc.) | □ | □ | □ | □ |
| Using online communication tools (e.g. social networking, online communities, online chat) | □ | □ | □ | □ |
| Using technology to support services to access and use your health and social care resources | □ | □ | □ | □ |
| Using the Internet safely and securely | □ | □ | □ | □ |
| Understanding issues of confidentiality and data protection | □ | □ | □ | □ |
| Accessing online learning opportunities | □ | □ | □ | □ |
| Other | □ | □ | □ | □ |

*□ Other (please specify)*

**About you**

1. How old are you? (Please tick one)
   - *18-29*
   - *30-39*
   - *40-49*
   - *50-59*
   - *60-69*
   - *70+*
2. Which of the following best describes how you think about your gender identity? (Please tick one)
   - *Male*
   - *Female*
   - *Non-binary*
   - *Other*
   - *Prefer not to say*
3. What is the highest level of education you have completed? (Please tick multy)
   - *School*
   - *College*
   - *University*
   - *Other*
4. Which of these responses best describes your living situation? (Please tick one)
   - *I live alone*
   - *I live only with my partner*
   - *I live with wider family members (e.g., sister, parent)*
   - *I live with people I am not related to (e.g., friends, hostel accommodation)*
   - *Prefer not to say*
   - *Other (please specify)*
5. What is your current situation regarding drug and alcohol use? (Please tick all that apply)
   - *I am currently using (non-prescribed) drugs*
   - *I am in treatment for problem substance use*
   - *I am prescribed medication for problem substance use*
   - *I use alcohol on a regular basis*
   - *I am not using drugs*
   - *I am not using alcohol*
   - *I am not in treatment*
   - *I am in recovery*
   - *Other*

*if selected other, please specify:*

**Evaluation Survey**

**For Service providers**

**Personal Information sheet**.

1. You are eligible to take part in the study if you are a professional involved in non-NHS service to people who use drugs or their family in Scotland and received a digital technology-based innovation funded by the Digital Lifelines Scotland programme and you do not work for an NHS organisation. Does this describe you?
   - *Yes/no (go to end of survey)*

**Consent form**

1. Do you consent to the above statements?
   - *Yes/no (go to end of survey)*

**Online Survey (Service Providers)**

**<< Organisation >>**

1. What type of organisation do you work in?
   - *Voluntary*
   - *Third Sector/Charity*
   - *Local Authority*
   - *other (please specify)*
2. What is the geographical scope of your service?
   - *Local (town/city/Local Authority level)*
   - *Regional (Health Board level),*
   - *National*
   - *Other (please specify)*
3. What is the main focus of your service for people who use drugs with complex needs?
   - *Harm reduction,*
   - *recovery support, treatment,*
   - *homelessness support,*
   - *a combination of supports,*
   - *other (please specify)*

Free text option to provide more information if needed

1. Please describe your current role in your organisation. How does it relate to people who use drugs with complex needs?
   - *Manager*
   - *Outreach worker*
   - *Recovery/harm reduction worker*
   - *Administrator*
   - *Supporter*
   - *Other(please specify)*

**<< Digital Literacy >>**

1. Do you have access to digital technology to allow you to undertake the following tasks?

|  | Yes | No | Not Applicable |
| --- | --- | --- | --- |
| A work email address so you can send and receive work communications | ○ | ○ | ○ |
| Access to online employee management systems (for example systems to record annual leave, absence, payroll etc.) | ○ | ○ | ○ |
| Internet connectivity | ○ | ○ | ○ |
| Access to technology for learning at work (e.g. online learning modules, tutorials, webcasts, videos) etc | ○ | ○ | ○ |
| Other |  |  |  |

- - *Other (please specify)*

1. Please tell us about your skills in using digital technology. Tick one box to show how much you agree with the following statements

| **I am confident that I can ...** | **Agree** | **Neutral** | **Disagree** | **Not Applicable** |
| --- | --- | --- | --- | --- |
| Use technology within the workplace | ○ | ○ | ○ | ○ |
| Log into PC or laptop at work | ○ | ○ | ○ | ○ |
| Send and receive work emails | ○ | ○ | ○ | ○ |
| Use software to help me in my role – Excel, Word, PowerPoint etc. | ○ | ○ | ○ | ○ |
| Share documents online with colleagues e.g. sending documents as attachments | ○ | ○ | ○ | ○ |
| Download and save documents from the Internet | ○ | ○ | ○ | ○ |
| Download and save documents from the intranet | ○ | ○ | ○ | ○ |
| Find the information I need on the Internet at work | ○ | ○ | ○ | ○ |
| Complete my mandatory training online | ○ | ○ | ○ | ○ |
| Use social media for work | ○ | ○ | ○ | ○ |
| Find relevant information using the internet to help with work | ○ | ○ | ○ | ○ |
| Understand internet safety and security | ○ | ○ | ○ | ○ |
| Understand the principles of online confidentiality and data protection | ○ | ○ | ○ | ○ |
| Participate in video conferences | ○ | ○ | ○ | ○ |
| Participate in webinars | ○ | ○ | ○ | ○ |
| Easily find online learning resources to help me in my role | ○ | ○ | ○ | ○ |
| Other |  |  |  |  |

- - *Other (please specify)*

1. Do you use the following at home?

|  | Yes | No |
| --- | --- | --- |
| PC | ○ | ○ |
| Laptop | ○ | ○ |
| Mobile phone with internet access (smartphone) | ○ | ○ |
| Mobile phone with no internet access | ○ | ○ |
| Tablet or iPad | ○ | ○ |
| Internet | ○ | ○ |

1. Please tell us what training you would welcome in relation to digital technology

|  | Most Important | Quite Important | Not  Important | Not  Applicable  I can do this  already. |
| --- | --- | --- | --- | --- |
| Getting started with computers (Logging onto computer, keyboard and mouse skills) | ○ | ○ | ○ | ○ |
| Computing basics (word processing, emails, spreadsheets) | ○ | ○ | ○ | ○ |
| Using the Internet (searching etc.) | ○ | ○ | ○ | ○ |
| Using online collaboration tools (e.g. social networking, online communities, online chat) | ○ | ○ | ○ | ○ |
| Using technology to support service users/clients/patients to access and use health and social care resources | ○ | ○ | ○ | ○ |
| Creating and publishing online content (e.g. blogs, podcasts) | ○ | ○ | ○ | ○ |
| Participating in videoconferences and webinars | ○ | ○ | ○ | ○ |
| Using the internet safely and securely | ○ | ○ | ○ | ○ |
| Understanding issues of confidentiality and data protection | ○ | ○ | ○ | ○ |
| Accessing online learning opportunities | ○ | ○ | ○ | ○ |
| Other | ○ | ○ | ○ | ○ |

- - *Other (please specify)*

**<<Clients>>**

1. Please estimate what proportion of your clients who use drugs have access to internet-connected technology?

|  | All of them | More than half | Approximately half | Less than half | A very small minority | I don’t know |
| --- | --- | --- | --- | --- | --- | --- |
| Smartphone | ○ | ○ | ○ | ○ | ○ | ○ |
| Laptop | ○ | ○ | ○ | ○ | ○ | ○ |
| Personal computer (PC) | ○ | ○ | ○ | ○ | ○ | ○ |
| Tablet | ○ | ○ | ○ | ○ | ○ | ○ |
| Other | ○ | ○ | ○ | ○ | ○ | ○ |

Free text option if required.

1. In your opinion, what are the barriers for those who do not have a device or do not use digital technology? Please tick all that apply.
   - *Cannot afford to buy a device*
   - *cannot afford data packages*
   - *has lost their device or had it stolen and not replaced it*
   - *lack of trust in digital technology*
   - *has no need for the technology*
   - *does not have support or training to use devices*
   - *other (Please specify)*

We would welcome if you can expand on your answer here. Free text option to provide more details.

1. Does your organisation provide *information* to people who use drugs via digital technology (e.g. website, text messaging, app, other)? If so, please note how this information is provided and with what level of engagement?

|  | Well used | Somewhat used | Rarely used | Never used |
| --- | --- | --- | --- | --- |
| Website | □ | □ | □ | □ |
| Text messaging | □ | □ | □ | □ |
| Mobile app | □ | □ | □ | □ |
| Social media activity | □ | □ | □ | □ |
| Other | □ | □ | □ | □ |

- - If uptake is poor or could be better, please provide your view as to why that might be and ways to address this.
    - Free text box

1. Does your organisation provide *services* to people who use drugs via digital technology (e.g. online meetings with clients, phone call/text check-ins with clients, other)?
   - *If yes, please tick all that apply*
     - *One to one online support meeting,*
     - *Therapeutic groupwork,*
     - *Access to video call/consultation for specific issue,*
     - *Online booking for a service,*
     - *Phone call/text check in,*
     - *Online access to personal data record/history,*
     - *Other (please specify)*
   - *If no, do you know why? Please tick all that apply.*
     - *Lack of technology,*
     - *Lack of training in using technology,*
     - *Lack of data access,*
     - *Unfamiliarity with technology,*
     - *other (please specify) Please elaborate in the box below.*
2. Do you use digital technology to share information regarding support for clients with other organisations?
   - Yes. Please provide information on what this support looks like and what other organisations are provided with information
   - No. Please use the text box to let us know why not (data protection, privacy concerns, lack of time, have not thought about it…)
3. Do other organisations share information on their clients and services with you? Yes/No

*If yes, what kind of information? (*Free text box)

- - *If not, what barriers are there? (*Free text box)

1. Would you welcome more training on using digital technologies in your work with people who use drugs with complex needs?

Yes/ No

Free text box for more information

If yes, what training would help you in using digital technology to provide support to clients? *(please specify)*

1. What training is needed to enable your clients to use digital technology/devices/software?

1. From your experience what support settings would benefit most from deploying digital technology?
   - *Homeless hostel/supported accommodation*
   - *Prison/police custody*
   - *Client’s home*
   - *Remote/ rural locations*
   - *NHS Service*
   - *Local Authority services*
   - *Voluntary sector services*
   - *Other*

1. In your opinion, what would make the most difference to the lives of people who use drugs in relation to access to/use of digital technologies to reduce harms in their lives. (Please tick all that apply)

- *Easy access to information on drug use and harm reduction*
- *Easy access to information on different types of treatment*
- *Easy access to information on services available locally*
- *Connection to support workers including peer support*
- *Connection to social support network e.g. Family*
- *Use of monitors to detect overdose*
- *Information on other health conditions*
- *Other (text box)*

1. Thinking about the examples you gave above in the survey so far, what are the *benefits/challenge* to your **organisation** from the delivery of ***information*** to clients via digital technologies/devices?

1. Thinking about the examples you gave in the survey so far, what are the *benefits/challenges* to your **organisation** from the delivery of ***services*** to clients via digital technologies/devices?

1. Thinking about the examples you gave above, what are the *benefits*/*challenges* to your **clients** from the delivery of ***information*** via digital technologies?

1. Thinking about the examples you gave above, what are the *benefits*/*challenges* to your **clients** from the delivery of ***services*** via digital technologies?

1. Is there anything else relevant that you would like to add that you have not been asked about above?

***<<Personal Information>>***

1. How long have you worked in your present job?
   - Under 1 year
   - 1 – 3 years
   - 4 – 5 years
   - 6 – 10 years
   - 11 – 20 years
   - 21 – 30 years
   - More than 30 years
2. How old are you?
   - *18-29,*
   - *30-39,*
   - *40-49,*
   - *50-59,*
   - *60-69,*
   - *70+*
3. Which of the following best describes how you think of your gender identity?
   - *Male*
   - *Female*
   - *Non-binary*
   - *Other*
   - *Prefer not to say.*
